# Major salivary gland carcinoma in KSA: A 10-year nationwide retrospective study of 571 cases

**DOI:** 10.1016/j.jtumed.2023.03.010

**Published:** 2023-04-02

**Authors:** Abdulaziz AlSalem, Mohammad AlKraidees, Abdullah AlKarni, Buthaina Yahya, Rana AlRamyan, Sultan AlSumairi, Mohammed AlEssa, Mohammed Elkrim

**Affiliations:** a Division of Otolaryngology-Head and Neck Surgery, Department of Surgery, King Abdulaziz Medical City, Ministry of National Guard Health Affairs, Riyadh, KSA; b College of Medicine, King Saud bin Abdulaziz University for Health Sciences, Riyadh, KSA; c Department of Otolaryngology-Head and Neck Surgery, College of Medicine, King Saud University, Riyadh, KSA; d King Abdullah International Medical Research Center, Riyadh, KSA

**Keywords:** أورام الغدد اللعابية, علم الأوبئة, ورم, المملكة العربية السعودية, معدل الإصابة, Epidemiology, Incidence rate, KSA, Neoplasm, Salivary malignancies

## Abstract

**Objectives:**

Major salivary gland carcinoma (MSGC) comprises a morphologically diverse group of rare tumours with different clinical behaviours, and epidemiology findings in the literature substantially vary by geographic location. The aim of this study was to conduct a comprehensive analysis of the incidence rates, anatomical sites, and histological subtypes of different salivary gland malignancies in the population of KSA.

**Methods:**

This retrospective cohort study included patients diagnosed with MSGC in KSA from 2008 to 2017, on the basis of the demographic characteristics and histological data retrieved from the Saudi Cancer Registry database. Malignant lesions were identified according to the International Classification of Diseases for Oncology, Third Edition (ICD-O-3) codes.

**Results:**

Salivary gland malignancies were diagnosed in 571 patients (50.10% males and 49.90% females) over the course of 10 years. The parotid gland was the site of origin in 69.9% of cases. The most common histological type was mucoepidermoid carcinoma (29.1%). Over a decade, the incidence rate ranged from (0.15–0.24) per 100,000 inhabitants. The peak incidence of salivary gland malignancies was observed in the fourth, fifth, and sixth decades of life (17.5%, 18.2%, and 16.8%, respectively).

**Conclusion:**

Compared with that in other parts of the world, the incidence of MSGC is significantly lower in KSA, with 0.15–0.24 cases per 100,000 people each year. However, the clinical manifestations of carcinoma of the salivary glands in KSA are similar to those described worldwide.

## الملخص

**أهداف البحث:**

يتألف سرطان الغدد اللعابية الرئيسي من مجموعة متنوعة شكليا من الأورام النادرة ذات السلوكيات السريرية المختلفة، وتتباين الدراسات حول علم الأوبئة في الابحاث اختلافا كبيرا اعتمادا على الموقع الجغرافي. الهدف من هذه الدراسة هو إجراء تحليل شامل لمعدلات الإصابة والمواقع التشريحية والأنواع الفرعية النسيجية لأورام الغدد اللعابية المختلفة في سكان المملكة العربية السعودية.

**طريقة البحث:**

تضمنت دراسة استعادية استعادية المرضى الذين تم تشخيص إصابتهم بسرطان الغدد اللعابية الرئيسية الأولية في المملكة العربية السعودية من عام 2008 إلى عام 2017، بناء على الخصائص الديموغرافية والبيانات النسيجية المسترجعة من قاعدة بيانات السجل السعودي للسرطان. تم تحديد الآفات الخبيثة باستخدام رموز التصنيف الدولي لأمراض الأورام، الإصدار الثالث.

**النتائج:**

تم تشخيص أورام الغدد اللعابية في 571 مريضا فوق 10 سنوات (50.10٪ ذكور و 49.90٪ إناث). كانت الغدة النكفية هي موقع المنشأ في 69.9٪ من الحالات. كان النوع النسيجي الأكثر شيوعا هو سرطان الجلد المخاطي (29.1٪). على مدى عقد، تراوح معدل الإصابة من (0.15 إلى 0.24) لكل 100000 نسمة. لوحظ ذروة الإصابة بأورام الغدد اللعابية في العقد الرابع (17.5٪) والخامس (18.2٪) والسادس (16.8٪) من العمر.

**الاستنتاجات:**

مقارنة بأجزاء أخرى من العالم، فإن معدل حدوث سرطان الغدد اللعابية أقل بشكل ملحوظ في المملكة العربية السعودية ، مع 0.15 إلى 0.24 حالة لكل 100000 شخص كل عام. ومع ذلك، فإن المظاهر السريرية لسرطان الغدد اللعابية كانت مماثلة لتلك الموصوفة في جميع أنحاء العالم.

## Introduction

Primary salivary gland tumours (SGT) are a morphologically diverse group of neoplasms that may present considerable management challenges to surgeons and oncologists. The global incidence rate of SGTs ranges from 0.4 to 13.5 per 100,000 people per year. Approximately 80% of all SGTs are benign, and malignant SGTs are very rare, with reported incidences of only 0.9–2.6 cases per 100,000, representing less than 5% of all head and neck malignancies.^1^ According to published data, the median age of diagnosis is 47, and the sex distribution is equal.^2^ Furthermore, the rarity and diversity of salivary gland carcinomas hinder epidemiological studies.

The 2017 World Health Organization classification comprises 31 SGT subtypes—11 benign and 20 malignant—constituting approximately 0.3% of all human tumours and 1–7% of all head and neck tumours.^3^ However, the rarity and heterogeneity of these salivary gland neoplasms make their diagnosis problematic. In addition, the anatomical location in which salivary gland malignancies develop varies according to multiple factors, including age, sex, race, and geographic area. These factors contribute to the development of different malignant histopathological subtypes.^4^

Interestingly, a study conducted in India by Subhashraj et al. has indicated a male predominance in the incidence of both benign and malignant tumours; moreover, benign tumours tend to evolve earlier in life and peak in the 5th decade of life, whereas malignant tumours tend to evolve later and peak in the 6th decade of life.^5^

In the Middle East, a descriptive study conducted in Jordan has indicated that malignant salivary gland neoplasms account for 6% of all malignant neoplasms of the head and neck. In the study, 51% of SGTs were located in the parotid gland, 21% were located in the submandibular gland, and 28% were located in the minor salivary glands. Thus, the absence of sublingual tumours confirms the rarity of tumour development in the Middle East compared with Western countries. In contrast to many other studies worldwide, the study reported a female predominance, with a male-to-female ratio of 1:1.2.^6^

Given the limited knowledge and data, understanding of the risk factors, incidence rate, age and sex distribution for malignant SGT in KSA is insufficient. To our knowledge, related studies are particularly lacking in the Middle East. Therefore, this study was aimed at providing data for comparison with other studies worldwide, to gain a better understanding of the characteristics of these tumours. Incidence rate, age, sex distributions, site of origin, mortality, and histological type, including grading and staging, were assessed in malignant SGTs in KSA.

## Materials and Methods

### Design and setting

The study was a nationwide epidemiological retrospective cohort study including patients diagnosed with primary major salivary gland carcinoma (MSGC) from 1 January 2008 to 31 December 2017, according to the STROBE guidelines published by the international EQUATOR association.

### Data sources, participants, and variables

The data were obtained from the Saudi Cancer Registry (SCR), which collects tumour data from all private, military, and Health Ministry hospitals in KSA through five regional offices., Data analysis and periodic reporting are conducted at the headquarters in Riyadh. The information in the SCR databases includes medical data and demographic characteristics of the patients, particularly sex, age, place of residence, tumour site of origin, histological subtype, tumour behaviour, tumour grade, tumour extension, tumour laterality, the basis for the diagnosis, and survival status. Diagnoses are coded according to the International Classification of Diseases for Oncology, Third Edition (ICD-O-3). The group under study was defined as patients diagnosed with MSGC between 2008 and 2017. In addition, the “summary stage” codes from the Surveillance, Epidemiology, and End Results (SEER) database were used to classify the extent of tumour spread.

### Statistical analysis

Data analysis was performed in the Statistical Package for the Social Sciences, SPSS version 23. Frequencies and percentages were used to display categorical variables. Mean, standard deviation, minimum, and maximum were used to present continuous variables. The chi-square test was used to test for associations across categorical variables. ANOVA was also used to test for association, and was followed by a Tukey post-hoc test to determine exact differences between groups. The threshold for statistical significance was set at p < 0.05.

## Results

### Demographic characteristics of patients

This study included 571 patients, of whom 285 (49.90%) were male, and 286 (50.10) were female. Table 1 displays the socio-demographic profile of the patients. The age was 46.53 + 19.62 years on average, ranging from 1 to 122 years. Moreover, 42 (7.4%) were of paediatric age. All patients were Saudi residents, except two (0.4%) participants from outside the country. The central region had the highest prevalence of malignant SGTs (196 participants; 34.3%), and was followed by the western region (176 participants; 30.8%).

**Table 1:** Socio-demographic profiles of the participants (n = 571).

Demographical characteristics	n	%
Sex		
Male	285	49.90
Female	286	50.10
Age group		
0–19 years	42	7.40
20–29 years	78	13.70
30–39 years	100	17.50
40–49 years	104	18.20
50–59 years	96	16.80
60–69 years	73	12.80
70 years or older	78	13.70
Place of residency		
Unknown	5	0.90
Central region	196	34.30
Eastern region	112	19.60
Northern region	23	4.00
Western region	176	30.80
Southern region	57	10.00
Out of the country	2	0.40
Age		
Mean	46.53	
Standard deviation	19.62	
Minimum	1	
Maximum	122	

### Age-specific incidence of major salivary gland carcinoma

**Figure 1:**
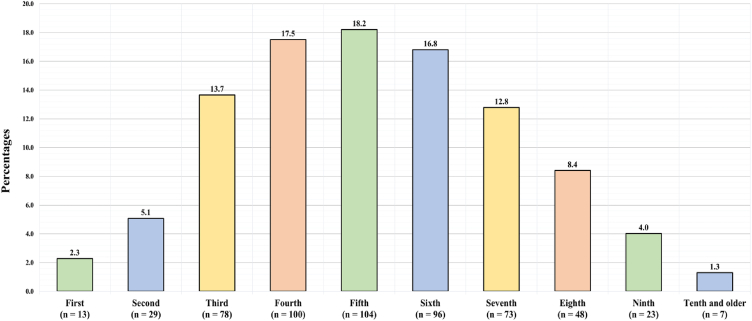
Tumors incidence across decades of life.

Figure 1 shows the incidence of MSGC by decade of life. The peak incidence of SGTs was observed in the fourth, fifth, and sixth decades of life (17.5%, 18.2%, and 16.8%, respectively). In contrast, the incidence rate was lowest in the first decade of life (2.3%), and in the tenth decade and beyond (1.3%).

### Tumour profile and morphology

Table 2 presents the tumour profiles of the participants. Most tumours were found in the parotid gland (n = 399, 69.9%), followed by the submandibular gland (18.9%) and the sublingual gland (1.8%). Regarding tumour morphology, mucoepidermoid carcinoma was the most common subtype (n = 166, 29.1%), followed by adenoid cystic carcinoma (n = 92, 16.1%), acinic cell carcinoma (n = 65, 11.4%), and adenocarcinoma not otherwise specified (NOS) (n = 60, 10.5%), and 188 (32.9%) had various other morphologies. A total of 271 (47.5%) patients had localized tumours, 67 (11.7%) had regional lymph node extension, and 57 (10%) had distant metastases. In addition, 273 (47.8%) of the participants had right lateralization, 228 (39.9%) had left lateralization, and 26 (4.6%) had paired site tumours (bilateral). In most cases (499; 87.4%), the diagnosis was based on the histology of the primary tumour.

**Table 2:** Tumour profile (n = 571).

Question	n	%
Primary site		
Parotid gland	399	69.9
Submandibular gland	108	18.9
Sublingual gland	10	1.8
Major salivary gland, non-otherwise specified	54	9.4
Morphology		
Mucoepidermoid carcinoma	166	29.1
Adenoid cystic carcinoma	92	16.1
Acinar cell carcinoma	65	11.4
Adenocarcinoma non-otherwise specified	60	10.5
Others	188	32.9
Extension		
Localized	271	47.5
Regional: direct extension	68	11.9
Regional: lymph node	67	11.7
Regional: dir ext and lymph node	31	5.4
Regional NOS	1	0.2
Distant metastasis	57	10
Unknown	76	13.3
Lateralization		
(Unknown)	44	7.7
Right	273	47.8
Left	228	39.9
Paired	26	4.6
Basis of diagnosis		
DCO (death certificate)	2	0.4
Medical imaging (X-ray, US)	1	0.2
Cytology/haematology	57	10
Histology of metastases	10	1.8
Histology of primary	499	87.4
Unknown	2	0.4
Patient status		
Alive	525	91.9
Dead	44	7.76
Unknown	2	0.4
Year of diagnosis		
2008	45	7.90
2009	63	11.00
2010	54	9.50
2011	52	9.10
2012	58	10.20
2013	64	11.20
2014	47	8.20
2015	52	9.10
2016	68	11.90
2017	68	11.90

NOS = non-otherwise specified.

### Incidence of salivary gland tumours per 100,000 inhabitants over time

**Figure 2:**
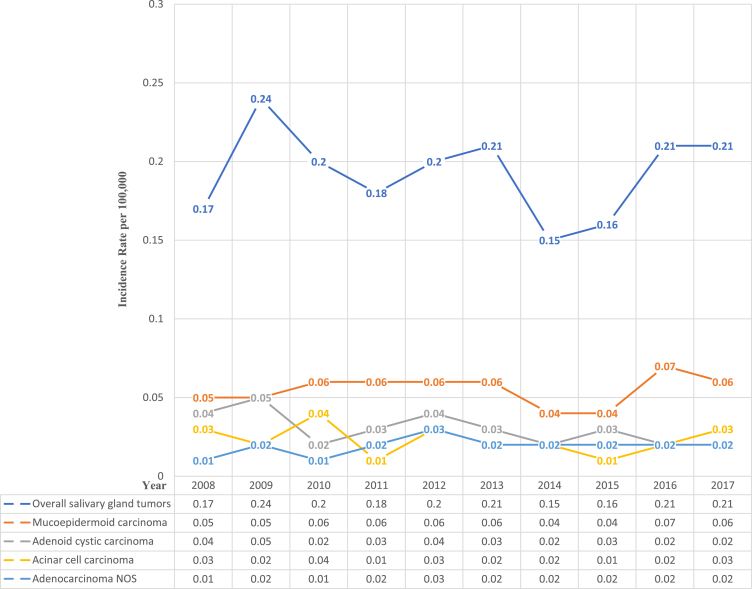
Incidence of salivary gland tumors per 100,000 by Year.

Figure 2 illustrates that the overall pattern of SGT incidence was irregular, lacked a clear pattern (in terms of increasing or decreasing over time), and displayed a fluctuating pattern. Both mucoepidermoid and adenoid cystic carcinoma showed a semi-consistent incidence pattern over time, whereas both acinar cell carcinoma and adenocarcinoma NOS showed an irregular fluctuating pattern. The highest incidence of SGTs (0.24 per 100,000) was observed in 2009, whereas 2014 had the lowest incidence (0.15 per 100,000). Table 2 demonstrates patient status: 525 (91.9%) were alive, 44 (7.76%) were dead, and 2 (0.4%) did not have a documented status. All patients who died had cancer-associated causes of death.

### Variations in participant and tumour characteristics by morphology

As shown in Table 3, age was significantly associated with MSGC type (p < 0.001). Tukey post-hoc test revealed that the patients with adenocarcinoma NOS were significantly older than those with mucoepidermoid carcinoma and acinar cell carcinoma (p < 0.05). Moreover, patients with adenoid cystic carcinoma were significantly older than those with acinar cell carcinoma (p < 0.05). Sex was also significantly associated with MSGC type (p = 0.005). Females had a significantly higher incidence of acinar cell carcinoma than males (22.4% vs 11%), whereas males had a significantly higher incidence of mucoepidermoid carcinoma than females (48.9% vs 38.3%). Although the parotid gland was by far the most common site of origin for all types of SGTs, a significant difference in the primary site of the tumour was observed across MSGC types (p < 0.001); acinar cell carcinoma had a relatively lower rate of the submandibular gland as the primary site, whereas adenoid cystic carcinoma had a relatively higher rate. No patients with adenoid cystic carcinoma had grade IV tumours (p < 0.001), whereas other types had varying rates of grade IV tumours. In addition, patients with adenocarcinoma NOS had a higher incidence of grade III tumours than patients with other types. MSGC type was also significantly associated with extension (p = 0.026) and mortality (p < 0.001): adenocarcinoma NOS had the highest rates of both metastasis and regional (direct and lymph node) extension, as well as the highest mortality rate. The correlation between lateralization and SGT type was not statistically significant.

**Table 3:** Variations in participant characteristics and tumour characteristics by morphology of major salivary gland malignancy.

Factor	Morphology				P-value
	Mucoepidermoid carcinoma	Adenoid cystic carcinoma	Acinar cell carcinoma	Adenocarcinoma NOS	
Age	41.81 + 18.32	47.47 + 16.12	38.49 + 18.46	54.5 + 17.63	<0.001^a^
Sex					0.005^a^
Male	89 (48.9%)	39 (21.4%)	20 (11%)	34 (48.7%)	
Female	77 (38.3%)	53 (26.4%)	45 (22.4%)	26 (12.9%)	
Primary site					<0.001^a^
Parotid gland	137 (82.5%)	53 (60.2%)	59 (90.8%)	43 (71.7%)	
Submandibular gland	17 (10.2%)	30 (34.1%)	2 (3.1%)	15 (9%)	
Major salivary gland, NOS	12 (7.2%)	5 (5.7%)	4 (6.2%)	8 (13.3%)	
Grade					<0.001^a^
Grade I (well differentiated)	36 (26.7%)	9 (31%)	11 (45.8%)	4 (12.1%)	
Grade II (moderately differentiated)	65 (48.1%)	15 (51.7%)	7 (29.2%)	8 (24.2%)	
Grade III (poorly differentiated)	18 (13.3%)	5 (17.2%)	3 (12.5%)	16 (48.5%)	
Grade IV (undifferentiated anaplastic)	16 (11.9%)	0 (0%)	3 (12.5%)	5 (15.2%)	
Extension					0.026^a^
Localized	89 (58.9%)	50 (60.2%)	36 (67.9%)	20 (40%)	
Regional: direct extension	19 (12.6%)	15 (18.1%)	5 (9.4%)	5 (10%)	
Regional: lymph node	24 (15.9%)	7 (8.4%)	6 (11.3%)	9 (18%)	
Regional: direct and lymph node extension	9 (6%)	2 (2.4%)	2 (3.8%)	4 (8%)	
Distant metastasis	10 (6.6%)	9 (10.8%)	4 (7.5%)	12 (24%)	
Lateralization					0.967
Right	81 (51.9%)	40 (45.5%)	31 (50%)	27 (49.1%)	
Left	68 (43.6%)	43 (48.9%)	29 (46.8%)	25 (45.5%)	
Paired	7 (4.5%)	5 (5.7%)	2 (3.2%)	3 (5.5%)	
Status					0.034^a^
Dead	10 (6.1%)	2 (2.2%)	1 (1.6%)	7 (11.7%)	
Alive	155 (93.9%)	90 (97.8%)	63 (98.4%)	53 (88.3%)	

a Significant at 0.05, NOS = non-otherwise specified.

## Discussion

The presented epidemiological analysis of major salivary gland malignancies in KSA provides extensive and comprehensive data from the past decade. Unfortunately, despite the tremendous advances in health care, little information is available on the clinical presentation of tumours of the head and neck in KSA.

During this 10-year study, we reviewed 571 patients with MSGC; tumours were found across all age groups (ages 1–122 years), and a similarly wide range has been reported in other studies.^5^^,^^7^, ^8^, ^9^ MSGC are extremely rare in children. Epidemiological research based on the North American population-based SEER database identified 12,834 cases registered from 1973 to 2006, with a 2% rate of paediatric cases across the entire study group.^10^ Similarly, low rates of 2–6% have been reported in studies from Turkey, India, and Brazil.^5^^,^^8^^,^^11^ In our study, a higher percentage of tumours was found in the paediatric age group, with 42 patients in the children and adolescents group and a 7.4% paediatric incidence rate. Moreover, on the basis of the analyses performed regarding the mean age of occurrence of various salivary gland pathologies, differences were observed according to lesion type and the years in the period analysed. For example, the mean age of patients with adenocarcinoma NOS was significantly greater than that of patients with mucoepidermoid carcinoma and acinar cell carcinoma (p < 0.05). Similarly, one study (Gao et al., 2017) has reported a high median age over 50 years for patients with adenocarcinoma NOS, salivary duct carcinoma, and squamous cell carcinoma.^7^

No sex predominance was observed: 285 (49.90%) cases were in males, and 286 (50.10%) cases were in females. In Canada and the United Arab Emirates, however, the MSGC incidence is higher among men, ranging from 59% to 61%.^12^^,^^13^ Nevertheless, our data indicated a significant association with MSGC type (p = 0.005): females had a higher rate of acinar cell carcinoma than males (22.4% vs 11%), and males had a higher rate of mucoepidermoid carcinoma than females (48.9% vs 38.3%).

In our study, the incidence of MSGC in the fourth, fifth, and sixth decades of life was 17.5%, 18.2%, and 16.8%, respectively, and peaked during the fifth decade. Other epidemiological studies have reported similar results.^8^^,^^12^^,^^14^ However, the highest incidence of mucoepidermoid and acinic cell carcinomas occurred in the third and fourth decades, in agreement with findings from other studies.^14^ The annual incidence rates of MSGC range from slightly less than 2 to greater than 0.05 per 100,000 population worldwide.^15^ During the past decade, the annual incidence of MSGC in KSA ranged between 0.15 and 0.24 per 100,000 people (Figure 2). Thus, malignant SGTs are relatively rare: in a population of 35 million, only 52–84 new cases of salivary gland cancer are expected each year (Table 2). This finding clearly calls for subspecialization in managing this disease, even within cancer centres. However, studies conducted on European, North, and South American populations have shown a higher incidence of malignancies and suggest geographic variation in the frequency of these tumours.^8^^,^^9^^,^^16^, ^17^, ^18^ In our study, the overall pattern of malignant SGT incidence was irregular, and no discernible pattern of increase or decrease over time was observed. In contrast to our findings, the national statistics for malignant parotid neoplasms in England (1997–2006) have indicated a rise in the number of cases from 1.1/100,000 in 1997 to 1.3/100,000 in 2006.^17^

According to the literature, the parotid gland is the most frequent anatomical site for MSGC.^5^^,^^8^^,^^9^^,^^12^^,^^16^ In the present study, the parotid gland was the most common primary tumour site and constituted (69.9%), or 36–58 new cases per year, and was followed by the submandibular and sublingual glands, which accounted for 18.9% and 1.8%, respectively. All large worldwide series of MSGC have shown a similar distribution.^5^^,^^8^^,^^9^^,^^12^^,^^16^ However, the sublingual gland involvement was significantly higher in studies from the UK, Africa, China, and the Middle East (5.6%, 6.5%, 12%, and 13.6%, respectively).^7^^,^^17^^,^^19^^,^^20^ Table 4 demonstrates the worldwide distribution of major salivary gland malignancies reported within the same period.

**Table 4:** Worldwide distribution of major salivary gland malignant tumours reported within the same period.

Country				Primary tumour site					
	Author	Study period	N of cases	Parotid (%)	Submandibular (%)	Sublingual (%)	Not otherwise specified (%)	Incidence (per 100,000)	Most common malignant tumours
KSA	Present study	2008–2017	571	69.9	18.9	1.8	9.4	0.15 to 0.24	MEC > ACC
China	Gao et al., 2017^7^	1963–2012	1430	70.2	17.8	12		NR	MEC > ACC
Turkey	Kizil et al., 2013^11^	1984–2012	97	82.5	16.5	1		NR	MEC > ACC = AC
Poland	Żurek et al. 2021^18^	2010–2019	6844	65.3	NR	NR		1.78	NR
Iran	Taghavi et al., 2016^19^	2000–2015	22	63.7	22.7	13.6		NR	MEC > AC
UK	Bradley et al., 2013^17^	1988–2007	108	71.3	23.1	5.6		0.67–0.98	MEC > ACC
Denmark	Westergaard-Nielsen et al., 2020^9^	1990–2015	1066	77.6	19.2	2.8	0.4	0.9–1.6	ACC > AcCC
Nigeria	Lawal et al., 2015^20^	1994–2013	77	74	19.5	6.5		NR	MEC > ACC
USA	Boukheris et al. 2009^16^	1992–2006	5370	79.4	15.8	1	3.8	1.2	MEC > SCC
India	Subhashraj et al. 2008^5^	1991–2006	203	79.3	19.7	1		NR	ACC > MEC
Brazil	da Silva, 2017^8^	1997–2017	371	78.7	18.1	3.2		2.1	MEC > ACC
United Arab Emirates	AlSarraj, 2015^12^	1998–2014	83	76	18	1	5	NR	MEC > ACC

Abbrev.: MEC = mucoepidermoid carcinoma; ACC = adenoid cystic carcinoma; AC = adenocarcinoma not otherwise specified; AcCC = acinic cell carcinoma; SCC = squamous cell carcinoma; NR = not reported.

Mucoepidermoid carcinoma was the most common histological type (29.1%), which was followed by adenoid cystic carcinoma (16.1%) and acinar cell carcinoma (11.4%). Mucoepidermoid carcinoma has been reported as the predominant histological subtype among different populations.^7^^,^^8^^,^^11^^,^^12^^,^^17^^,^^21^^,^^22^ However, research conducted in Denmark and India has revealed that adenoid cystic carcinoma is the most prevalent disease.^5^^,^^9^ Our data revealed that adenocarcinoma NOS was the histological type associated with the greatest mortality and spread of disease: 18–26% nodal metastases and 24% distant metastasis were observed, thus indicating high-grade tumours with imprecise clinical behaviour; the mortality rate was 11.7% (Table 3).^14^ Table 5 demonstrates the relative proportion of major salivary gland malignancies subtypes among studies worldwide.

**Table 5:** Relative proportion of the five major subtypes of major salivary gland carcinoma (MSGC) among studies worldwide within the same period.

Country	Author	MSGC morphology			
		MEC (%)	ACC (%)	AcCC (%)	AC (%)
KSA	Present study	29.1	16.1	11.4	10.5
China	Gao et al., 2017^7^	27.3	23.8	8.25	10
Turkey	Kizil et al., 2013^11^	17.5	13.4	7.2	13.4
Iran	Taghavi et al., 2016^19^	40.9	13.6	4.5	27.2
UK	Bradley et al., 2013^17^	27.7	24	11.1	20.3
Denmark	Westergaard-Nielsen et al., 2020^9^	11.9	20.4	13.5	9.7
Nigeria	Lawal et al., 2015^20^	44.1	31.1	6.4	9
USA	Boukheris et al. 2009^16^	28.9	13.2	14.3	11.9
India	Subhashraj et al. 2008^5^	19.2	20.1	8.8	10.8
Brazil	da Silva, 2017^8^	32.6	21	7.8	14.8
UAE	AlSarraj, 2015^12^	34.9	18.1	10.8	2.4

Abbrev.: MEC = mucoepidermoid carcinoma; ACC = adenoid cystic carcinoma; AcCC = acinic cell carcinoma; AC = adenocarcinoma not otherwise specified; NR = not reported.

The strength of this study is that, compared with other salivary gland carcinoma epidemiological studies in the literature, we describe one of the largest population-based cohorts from the Middle East. In this study, we analysed the epidemiology of MSGC among Saudis, on the basis of extensive and comprehensive data gathered over a decade, which may be used in the prognosis and planning of medical services and serve as a foundation for future research. To our knowledge, this is the largest national series of MSGC. Moreover, we compared our data with other worldwide series. In addition, this study demonstrated that Saudis have a lower incidence of MSGC than the rest of the world, thus suggesting that further research is warranted to investigate whether environmental, behavioural, and genetic factors have roles in determining MSGC risk.

The study was limited by its retrospective design and some limitations associated with the lack of clinical data, such as symptomatology, stage of malignant lesions, and treatment, which are not reported in the SCR and thus could not be included in the study. The reason for these limitations is that the SCR database data are recorded primarily for administrative purposes rather than research.

## Conclusion

This retrospective study of MSGC showed that KSA had a relatively low incidence rate, with only 0.15 to 0.24 cases per 100,000 person-year, with respect to those in other parts of the world. Thus, management in highly specialized cancer treatment centres is required. Although MSGC is rare in adults and highly infrequent in children, Saudi children/adolescents have a slightly higher incidence rate than that reported in the literature. The rest of our results are consistent with those reported by previous population studies. Furthermore, continuous studies reporting the incidence and characteristics of these lesions will be essential to keep physicians and surgeons updated and have a low detection threshold, thus substantially influencing tumour prognosis.

## Source of funding

This research did not receive any specific grant from funding agencies in the public, commercial, or not-for-profit sectors.

## Conflict of interest

The authors have no conflict of interest to declare.

## Ethical approval

Ethics approval was exempted by The Institutional Review Board (IRB) of King Abdullah International Medical Research Center (KAIMRC), Riyadh, KSA (date of approval 3 August 2021, reference number NRC21R/085/03).

## Consent

The need for informed consent was waived, because no information identifying individual people was included.

## Authors contributions

Concept and design: AAA and MSA. Literature search, data acquisition, data analysis, statistical analysis, interpretation of data, and initial drafting of the manuscript: MSA, AFA, BJY, RKA, and SAA. Project administration: MSA and AFA. Revision, editing, and drafting of the final manuscript: AAA. Critical revision of the manuscript for important intellectual content: AAA, MAA, and MME. All authors were involved in critically revising the manuscript for important intellectual content. All authors gave final approval of the version to be published. All authors agree to be accountable for all aspects of the work. All authors have critically reviewed and approved the final draft and are responsible for the content and similarity index of the manuscript.
